# Severe bleeding associated with lumen-apposing metal stent placement for walled-off necrosis: bloody memory in WONderland

**DOI:** 10.1055/a-2335-6707

**Published:** 2024-06-25

**Authors:** Shuichi Tange, Tomotaka Saito, Yousuke Nakai, Tsuyoshi Hamada, Yusuke Watanabe, Naminatsu Takahara, Mitsuhiro Fujishiro

**Affiliations:** 1 Department of Gastroenterology, The University of Tokyo, Graduate School of Medicine, Tokyo, Japan; 2 26782 Department of Endoscopy and Endoscopic Surgery, The University of Tokyo Hospital, Tokyo, Japan; 3 Department of Radiology, The University of Tokyo, Graduate School of Medicine, Tokyo, Japan

Lumen-apposing metal stents (LAMSs) may facilitate endoscopic ultrasound (EUS)-guided treatment of walled-off necrosis (WON) as a transluminal port for necrosectomy 1 2 . However, this modality has a risk of potentially lethal bleeding 3 4 . In this case, we experienced dreadful bleeding when conducting EUS-guided LAMS placement with balloon dilation for direct endoscopic necrosectomy (DEN) ( Video 1 ).

Bleeding associated with endoscopic ultrasound-guided placement of a lumen-apposing metal stent for walled-off necrosis with balloon dilation.Video 1

A 41-year-old man presented with infectious WON due to severe biliary pancreatitis, and EUS-guided treatment was scheduled. No abnormal vessels were observed along the puncture route both on contrast-enhanced computed tomography (CT) ( Fig. 1 ) and Doppler endosonography. We placed a LAMS 15 mm in width (Hot AXIOS; Boston Scientific Japan, Tokyo, Japan) through the electrocautery-assisted transgastric puncture. To facilitate the endoscopic passage for DEN 5 , we dilated the saddle of the LAMS up to 15 mm using a balloon dilator (Giga2; Century Medical, Tokyo, Japan) ( Fig. 2 **a** ). At the end of DEN, a decent amount of blood clots was observed in the stomach, but there was only intermittent oozing around the flange of the LAMS. Subsequent CT revealed no extravasation, but the patient developed hemorrhagic shock with cardiopulmonary arrest five hours after the procedure. Repeated CT ( Fig. 2 **b** ) and subsequent angiography ( Fig. 2 **c** ) delineated an extravasation from the left gastric artery branch beside the LAMS, and hemostasis was achieved via vigorous coiling of the responsible artery ( Fig. 2 **d** ). The bleeding did not recur until WON resolution, and the LAMS was removed endoscopically without bleeding. The patient was discharged without any disability ( Fig. 3 ).

**Fig. 1:**
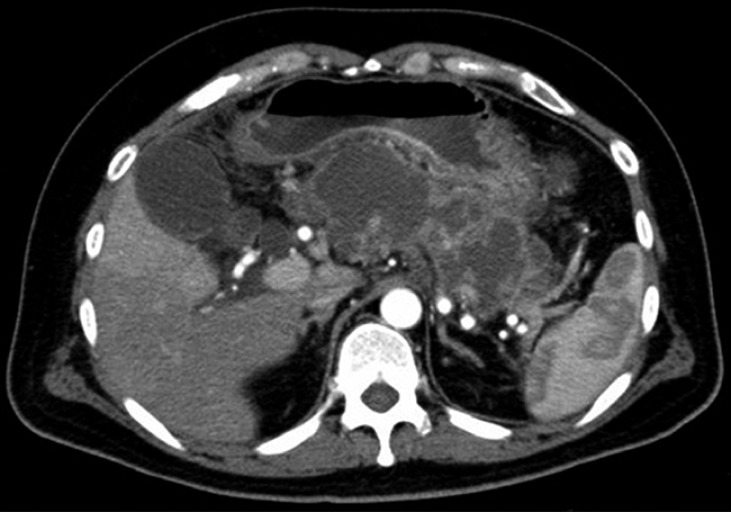
Computed tomography delineating a walled-off necrosis lesion. No abnormal vessels were observed along the possible puncture route of endoscopic ultrasound-guided puncture.

**Fig. 2:**
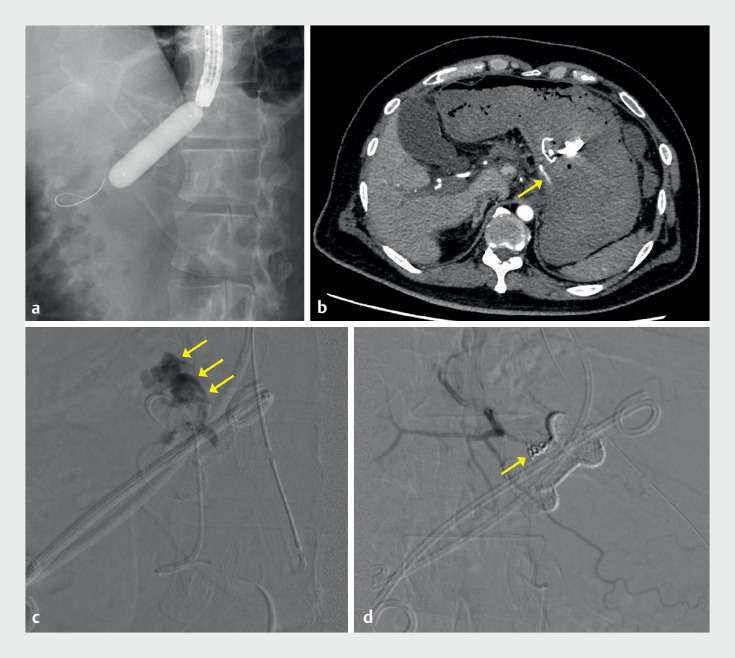
Severe bleeding associated with endoscopic ultrasound-guided placement of a lumen-apposing metal stent (LAMS) for walled-off necrosis (WON). **a** Fluoroscopy showing balloon dilation of the inner lumen of the LAMS for immediate necrosectomy. After the end of the necrosectomy, intermittent blood oozing was suspected in the area adjacent to the LAMS. **b** Computed tomography delineating contrast extravasation (arrow) along the lesser curvature of the stomach. **c** Emergent angiography delineating contrast extravasation from a branch of the left gastric artery (arrow). **d** Successful coiling (arrow) for hemostasis.

**Fig. 3:**
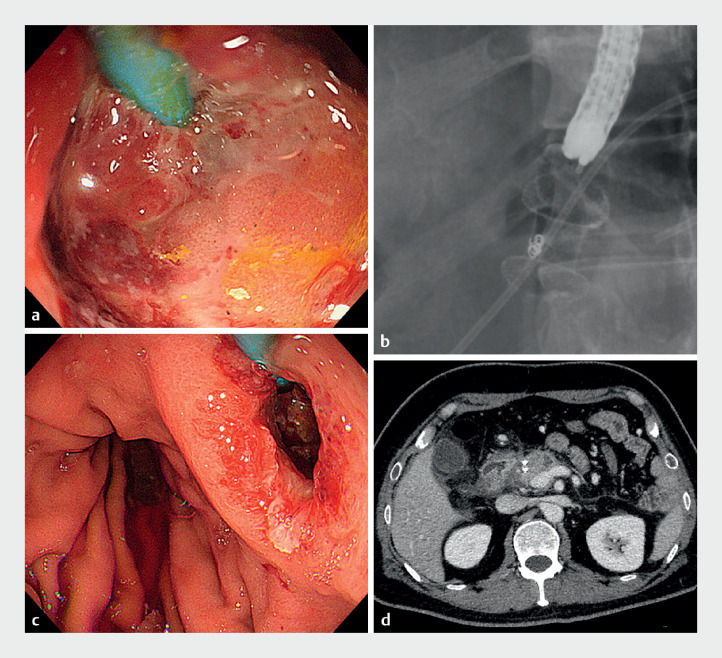
The completion of endoscopic treatment of WON without recurrence of bleeding. **a** Necrotic tissue within WON was removed by endoscopic necrosectomy. **b** Fluoroscopic image of LAMS removal. **c** Endoscopic image after LAMS removal without significant bleeding. **d** Computed tomography showed resolution of WON.

In this case with nearly fatal bleeding, severe bleeding was not predictable based on preprocedural imaging, highlighting the importance of watchful monitoring and support of radiologists and surgeons in case of serious complications after LAMS placement for WON. Balloon dilation of a LAMS may increase the bleeding risk, and the feasibility of this procedure should be investigated.

Endoscopy_UCTN_Code_TTT_1AS_2AJ

## Notes

Endoscopy E-Videos https://eref.thieme.de/e-videos
*E-Videos* is an open access online section of the journal *Endoscopy* , reporting on interesting cases and new techniques in gastroenterological endoscopy. All papers include a high-quality video and are published with a Creative Commons CC-BY license. Endoscopy E-Videos qualify for HINARI discounts and waivers and eligibility is automatically checked during the submission process. We grant 100% waivers to articles whose corresponding authors are based in Group A countries and 50% waivers to those who are based in Group B countries as classified by Research4Life (see: https://www.research4life.org/access/eligibility/ ). This section has its own submission website at https://mc.manuscriptcentral.com/e-videos .
